# Timing correlations between cerebellar interpositus neuronal firing and classically conditioned eyelid responses in wild-type and Lurcher mice

**DOI:** 10.1038/s41598-018-29000-w

**Published:** 2018-07-16

**Authors:** Juan C. López-Ramos, Zbynek Houdek, Jan Cendelín, Frantisek Vožeh, José M. Delgado-García

**Affiliations:** 1 Division of Neurosciences, Pablo de Olavide University, Seville, Spain; 2 Department of Pathophysiology, Faculty of Medicine in Pilsen, Charles University, Pilsen, Czech Republic; 3 Department of Biology, Faculty of Medicine in Pilsen, Charles University, Pilsen, Czech Republic; 4 Biomedical Center, Faculty of Medicine in Pilsen, Charles University, Pilsen, Czech Republic

## Abstract

Classical eyeblink conditioning is an experimental model widely used for the study of the neuronal mechanisms underlying the acquisition of new motor and cognitive skills. There are two principal interpretations of the role of the cerebellum in the learning of eyelid conditioned responses (CRs). One considers that the cerebellum is the place where this learning is acquired and stored, while the second suggests that the cerebellum is mostly involved in the proper performance of acquired CRs, implying that there must be other brain areas involved in the learning process. We checked the timing of cerebellar interpositus nucleus (IPN) neurons’ firing rate with eyelid CRs in both wild-type (WT) and Lurcher (a model of cerebellar cortex degeneration) mice. We used delay and trace conditioning paradigms. WT mice presented a better execution for delay vs. trace conditioning and also for these two paradigms than did Lurcher mice. IPN neurons were activated during CRs following the activation of the orbicularis oculi muscle. Firing patterns of IPN neurons were altered in Lurcher mice. In conclusion, the cerebellum seems to be mostly related with the performance of conditioned responses, rather than with their acquisition.

## Introduction

Classical eyeblink conditioning is a well-known and accessible experimental model for the study of associative learning in mammals. Nevertheless, there is still some debate regarding the brain structures where this learning is acquired and stored. Following the mathematical models of Marr^1^ (1969) and Albus^2^ (1971), the experimental studies of Thompson’s group^3–5^ were the first to assign to the cerebellum an essential role in classical eyeblink conditioning, whereas other authors persist in arguing that the cerebellar structures have a role in the proper performance of eyelid responses, but not in its new acquisition and storage^6–8^. Finally, other studies propose that plastic changes underlying eyeblink conditioning are distributed across several cerebellar and extracerebellar regions, resulting in a network performance^9^. Such hypothesis could be in accordance with those works that assign an important role in this type of associative learning to the motor^10^ and the prefrontal^11–13^ cortices, the hippocampus^14^, or the amygdala^15^.

Those experimental approaches used various techniques, ranging from the lesion of cerebellar structures to electrophysiological recordings of cerebellar cortical and nuclear neurons, as well as the use of mutant or genetically manipulated mice^16–22^. The different approaches offered contradictory results. For example, they described several features of the conditioned process in mice, different from those in other mammals, such as auditory startle reflexes and learned specific fear responses, which seem to be more prominent in mouse eyeblink^15^, and must be considered when working with this species, particularly when working with stressed and anxious animals, as are Lurcher mice^23^.

Lurcher mice carry a spontaneous gain-of-function mutation in a gene codifying for the glutamate receptor delta2 (GluRδ2)^24^. Mice carrying a null mutation of this gene suffer from ataxia and synaptic plasticity alterations for long-term depression^25^. Lurcher heterozygous mutants begin to lose Purkinje cells at postnatal day 10 (P10), and by P90 all Purkinje cells have been functionally lost. As a consequence, 75% of inferior olive cells and 90% of granular cells have also disappeared, with the subsequent reduction in cerebellar volume^26–31^. Purkinje cells are the sole output from the cerebellar cortex, and project mainly to cerebellar deep nuclei. These nuclei project to brainstem centers, and receive afferents from collaterals of mossy and climbing fibers projecting to the cerebellar cortex. However, cerebellar nuclei of Lurcher mutants are almost not affected by the mutation of the gene codifying for the GluRδ2 receptor, or at least have no structural problems^26^ or a minimum cell damage^32–34^. In accordance, Lurcher mice seem to be a good model for studying IPN neurons’ firing correlation with the normal and putatively impaired classically conditioned eyelid movement^35^.

Here, we have developed a head-restricted freely moving muffled system for the study of cerebellar IPN neurons’ firing rate during classically conditioned eyelid responses in behaving mice. To obtain a precise detection of eyelid movements, we have implemented a high-speed video analysis and a Hall-based magnetic distance measurement system. For classical conditioning analysis, and in order to prevent the high excitability effects of stress- and anxiety-related responses in Lurcher mice, we have taken into account not only CRs, but also their relationships with unconditioned responses (URs). Finally, to study the putative relationships between the firing rate of IPN neurons and CRs, we have implemented automatic analysis software, capable of detecting fine timing correlations between them.

## Results

### Identification of recorded IPN neurons

**Figure 1:**
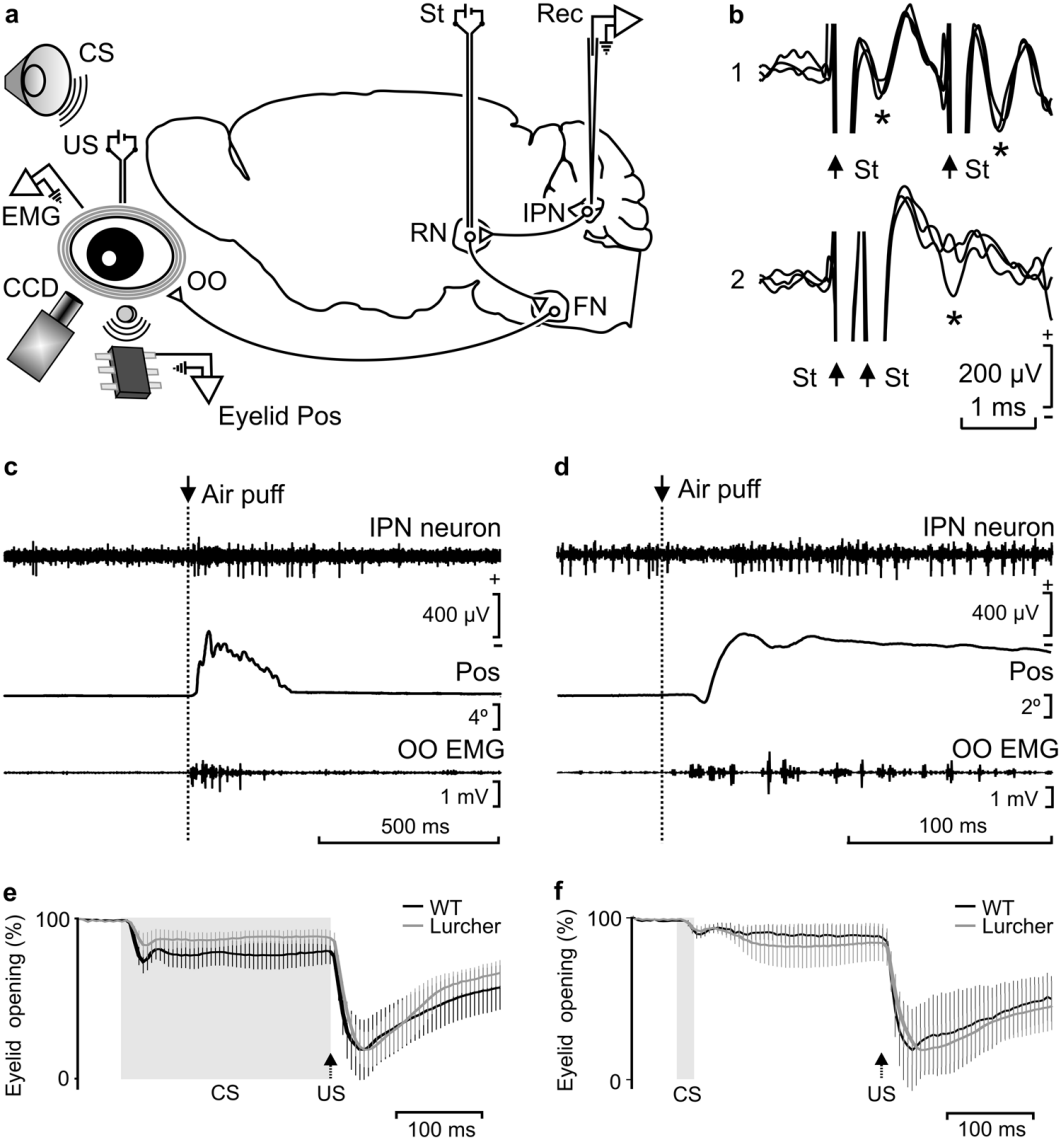
Experimental design and identification of recorded IPN neurons. (**a**) Diagrammatic representation of the experimental design. Mice were chronically implanted with stimulating electrodes on the left supraorbital nerve for US presentations and with EMG recording electrodes in the left orbicularis oculi (OO) muscle. A loudspeaker was used for the presentation of a tone as CS. Eyelid position (Eyelid Pos) was determined as the voltage difference between a Hall-effect sensor located on the head-holding system and a magnetic piece fixed to the lower eyelid. In addition, eyelid opening was detected with a fast CCD camera. Ipsilateral IPN neurons were recorded (Rec) with glass micropipettes and identified by their antidromic stimulation (St.) from the contralateral red nucleus (RN). FN, facial nucleus. (**b**) Overlapped (n = 3) traces of the antidromic activation (*) of a representative IPN neuron from the RN (St.) at threshold-straddling intensities and at different (1, 1.6 ms; and 2, 0.4 ms) interstimulus intervals. Note in 2 that the antidromic activation is partially prevented. Arrows indicate stimulus artifacts. (**c**,**d**) Representative examples of the firing rate of type A (**c**) and B (**d**) IPN neurons following the presentation of an air puff aimed at the ipsilateral cornea. Eyelid position (Pos) and OO EMG are also illustrated. (**e**,**f**) Typical CRs of trained WT and Lurcher animals collected during delay (**e**) and trace (**f**) conditioning paradigms. Values are mean ± SEM of eyelid closing percentages (n = 6 animals) obtained by a MATLAB-based analysis of the photographs taken with a fast CCD camera. Gray bands and black arrows represent CS duration and US presentation respectively. Note the different patterns of eyelid closing for delay and trace conditioning paradigms, but not between WT and Lurcher mice.

**Figure 2:**
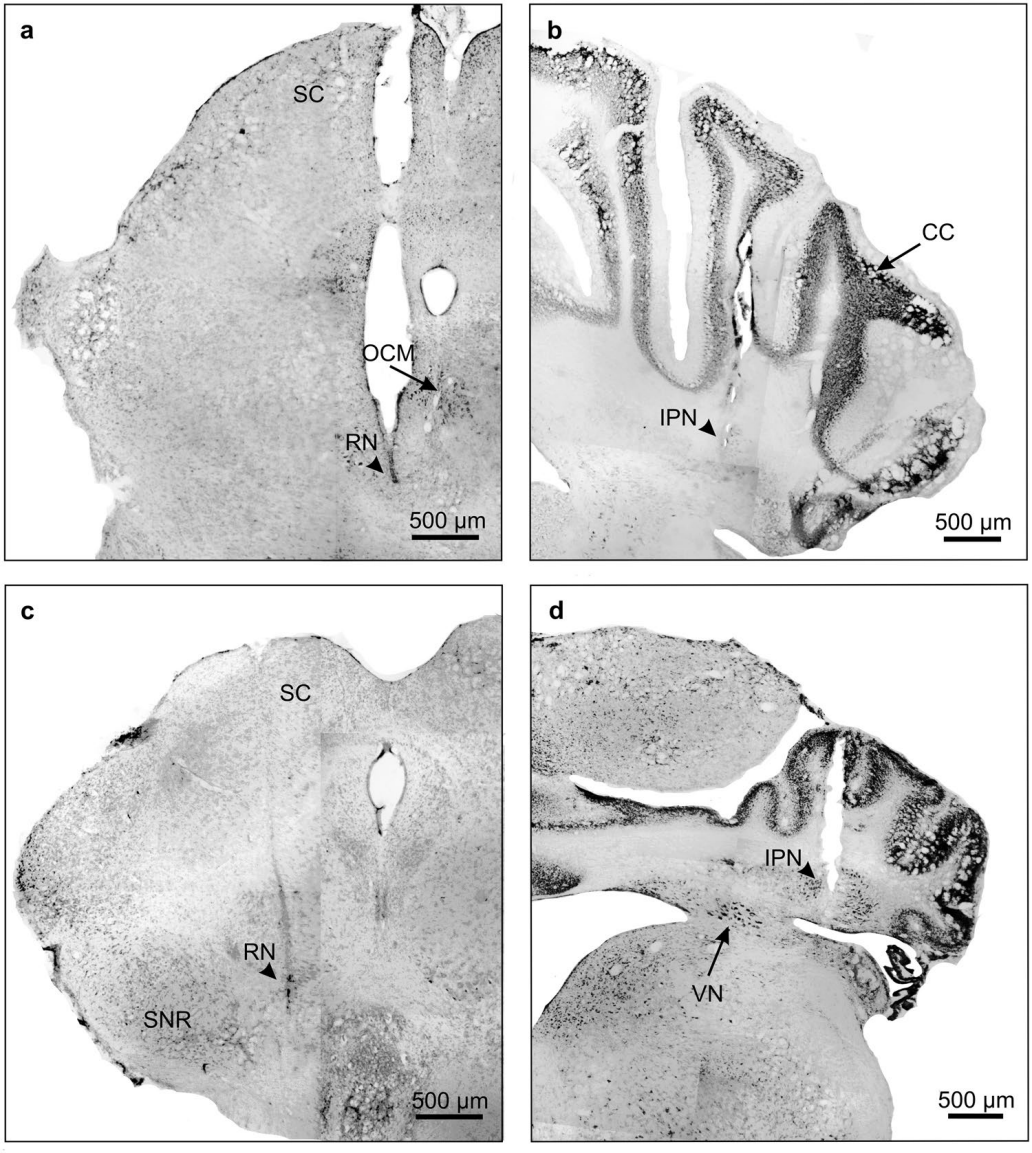
Photomicrographic reconstruction of recording and stimulating sites. Examples showing the tracks and end points of the tips (arrowheads) of the chronic electrodes used for red nucleus stimulation (**a**,**c**) and the glass micropipettes used for IPN neuron recording (**b**,**d**) in WT (**a**,**b**) and Lurcher (**c**,**d**) mice. Note the abnormal development of Lurcher mice cerebellar cortex (**d**). SC, superior colliculus; OCM, oculomotor nucleus; RN, red nucleus; SNR, substantia nigra; CC, cerebellar cortex; IPN, interpositus nucleus; VN, vestibular nucleus.

The IPN recording area was approached following a previous electrophysiological study^35^ and in accordance with a mouse stereotaxic atlas^36^. As illustrated in Fig. 1b, recorded neurons were identified by their antidromic activation from their projection site–i.e., the contralateral red nucleus–which was chronically implanted with stimulating electrodes just in the place where its activation evoked an identifiable eyeblink (see Fig. 2). The latency of the antidromic activation of IPN neurons in WT mice was 0.69 ± 0.01 ms (mean ± SEM, n = 29 recordings from n = 5 mice), while the latency for orbicularis oculi electromyographic (EMG) responses was 4.8 ± 0.2 ms (n = 20 recordings from n = 6 mice). Lurcher animals presented similar values for the antidromic activation of IPN neurons (0.70 ± 0.01 ms; n = 30 recordings; n = 6 mice) and for the initiation of the electromyographyc (EMG) activity of the orbicularis oculi muscle (4.9 ± 0.1 ms; n = 21 recordings; n = 6 mice).

With respect to their firing profiles and in accordance with previous descriptions^37^, two types of IPN neuron were recorded when air puffs were presented to the animal’s cornea and/or to tone presentations. Type A neurons were characterized by their activation by air-puff or tone presentations, with activation latencies of ≈10 ms for air-puff presentations, whereas type B neurons were characterized by their inactivation by the same stimuli (Fig. 1c,d). Because of their firing properties, type B neurons were not further considered in this study.

### Differences in CR patterns evoked by delay and trace paradigms

In order to characterize the displacement of the eyelid during CRs, 100 photographs per trial, obtained during the time lapse between 50 ms before and 450 ms after conditioned stimulus (CS) onset, were analyzed with the help of a custom-written MATLAB-based program. In Fig. 1e,f are represented the percentage (%) of eyelid displacement for CRs and URs obtained after the analysis of 100 conditioning trials [n = 6 animals per group (WT, Lurcher) and paradigm (delay and trace)]. As illustrated, there were no significant differences between WT and Lurcher animals. In contrast, it was evident there were different patterns of eyelid responses for delay (Fig. 1e) and trace (Fig. 1f) conditioning paradigms (see below).

### Firing rate of type A neuron during classical eyeblink conditioning

**Figure 3:**
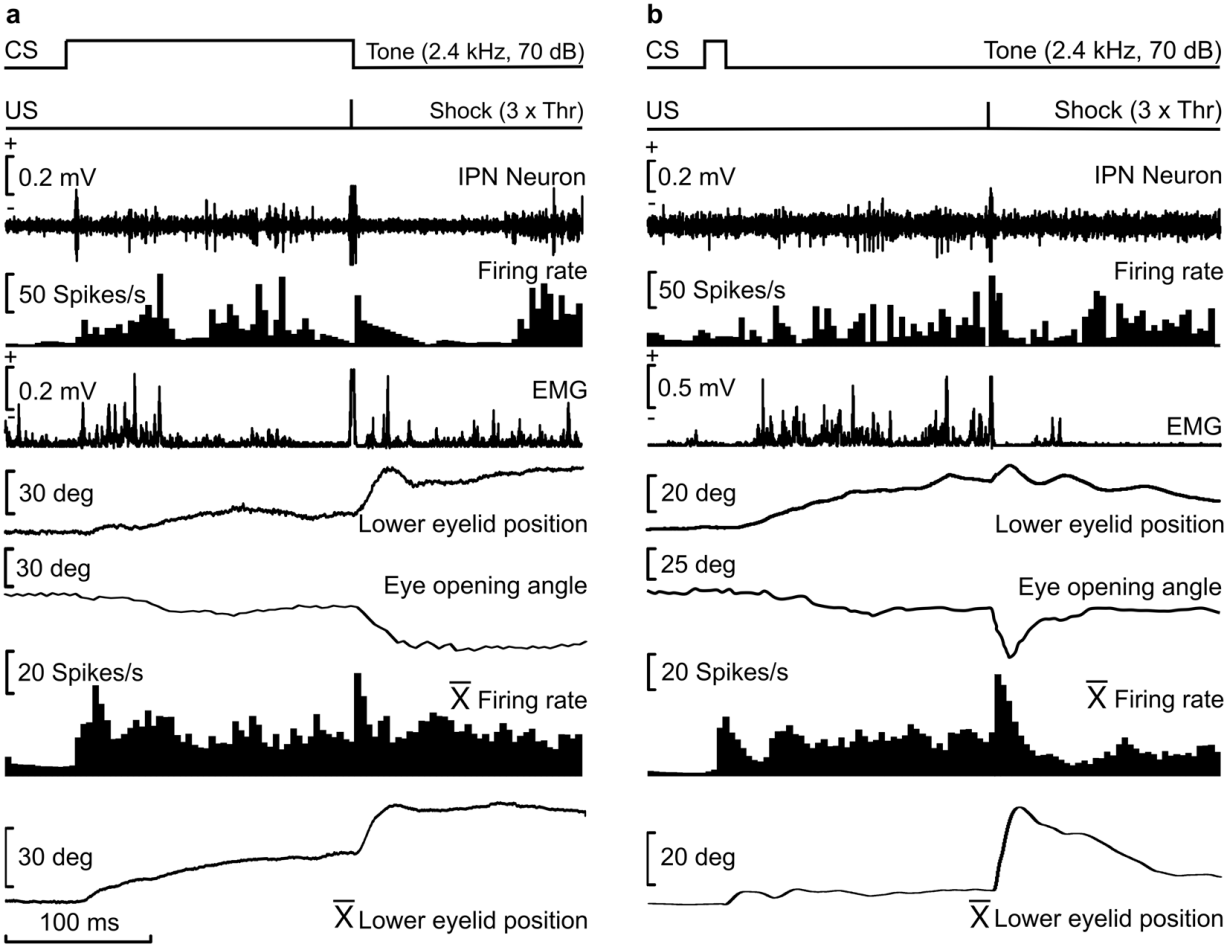
Firing activities of type A neurons during two different conditioning sessions using delay (**a**) or trace (**b**) conditioning paradigms. From top to bottom are represented the conditioning paradigm (CS and US presentations), the firing activity of the IPN neuron, the firing rate, the rectified EMG activity of the OO muscle, the lower-eyelid position detected on-line with the Hall-effect sensor, and the eye-opening angle detected off-line through a MATLAB-based analysis of photographs taken with a fast CCD camera. At the bottom are represented the averaged firing rate and the lower-eyelid position collected during the trials 41 to 60 of a single conditioning session.

As already described^35,38^ and further illustrated in Fig. 3a,b, type A neurons increased their firing rate following the initiation of the evoked CR. This burst of activity could be individualized for the successive CS and unconditioned stimulus (US) presentations or could be present throughout the CR. Some intermediate responses of type A neurons could also be observed.

As already reported for cats^39^, the kinematics of CRs was different during delay (Figs 1e and 2a) versus trace (Figs 1f and 2b) conditioning paradigms [F_(49,1029)_ = 4.49; *P* < 0.05], with significant differences of curve shapes (measured between 15 ms and 120 ms of the CRs; one-way ANOVA, n = 11 delayed and n = 12 trace response curves, *P* < 0.05). In addition, there were significant [F_(1,22)_ = 9.91; *P* < 0.05] differences between the firing rate of type A neurons averaged during CRs evoked by delay versus trace conditioning paradigms (one-way ANOVA, n = 12 delayed and n = 11 trace CRs). Single and averaged (Fig. 3a,b) firing profiles of type A neurons seemed to reproduce eyelid-position records–i.e., type A neuron firing was apparently related to eyelid position.

### Classical eyeblink conditioning of WT and Lurcher mice

**Figure 4:**
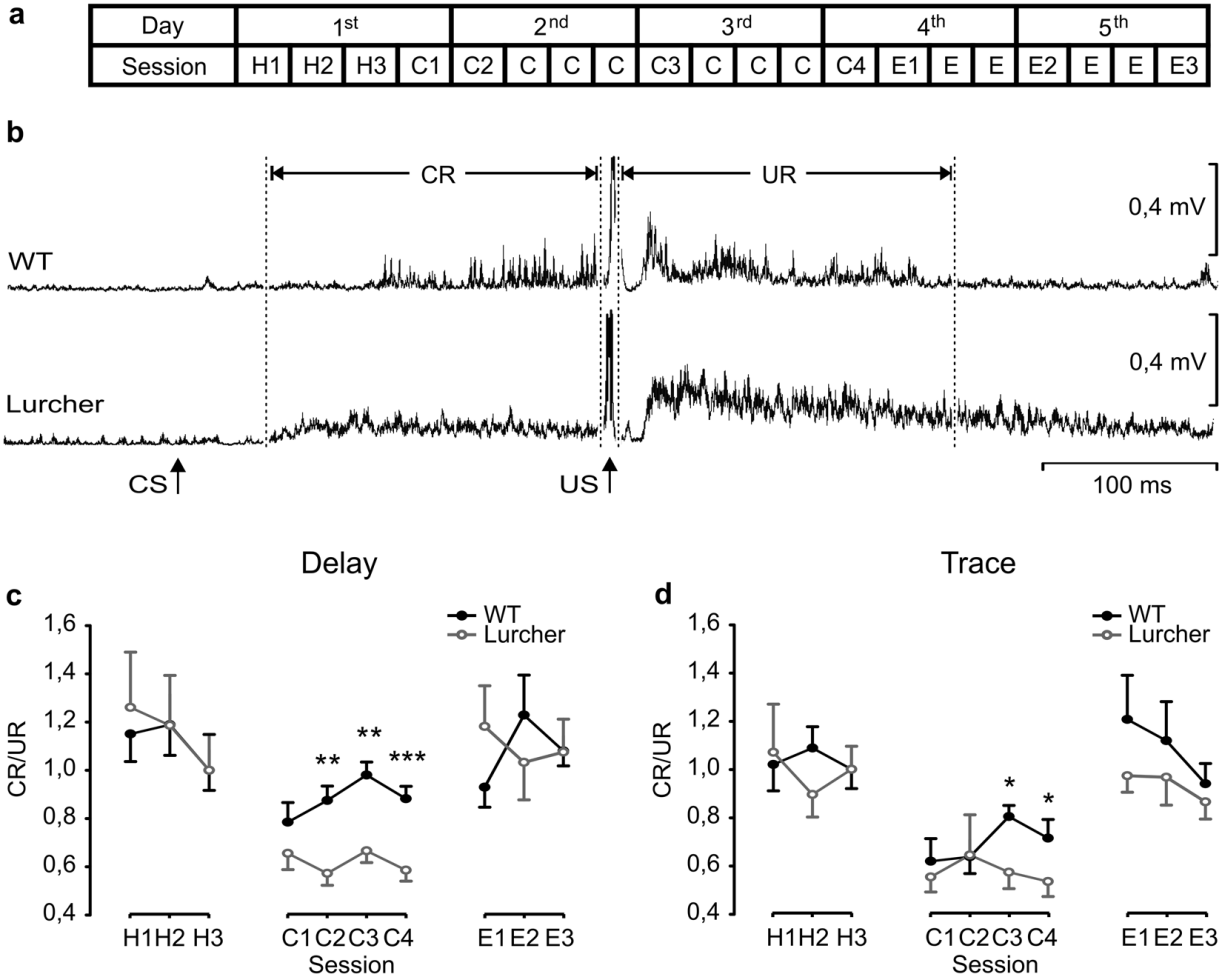
Evolution of classical eyeblink conditioning of WT and Lurcher mice. (**a**) Schematic representation of habituation (H), conditioning (C), and extinction (E) sessions, using both delay and trace conditioning paradigms. Each of the five (1st to 5th) training sessions consisted of 200 trials, divided in four sessions of 50 trials each. Sessions analyzed to obtain learning curves, shown in (**c**) and (**d**), are indicated by numbers (H1-H3, C1-C4, and E1-E3). (**b**) rEMG averaged from 50 trials from a C4 session of representative WT and Lurcher mice. CS and US presentations, and the CR and UR sections whose areas were quantified for the formulation of the learning curves shown in (**c**) and (**d**) are indicated. (**c**,**d**) CR/UR ratios of rEMG areas collected from WT and Lurcher mice during delay (**c**) and trace (**d**) conditioning paradigms. Represented data are mean ± SEM of the percentage (%) of CRs (n = 9 animals per group). *P < 0.05; **P < 0.01. Student-Newman-Keuls post hoc one-way ANOVA.

As illustrated in Fig. 4a, a classical conditioning task consisted of five 200-ms trials sessions, divided in 50 trials each, for both delay and trace paradigms^40^. We considered it interesting to determine the relationships between evoked CRs and URs (i.e., the conditioning performance index). CR/UR ratios were calculated from collected and rectified EMG (rEMG) recordings. During habituation and conditioning sessions, when the US was not presented, and no CR was evoked, the CR/UR ratios reached values around 1 (Fig. 4c,d). Thus, during habituation sessions, WT mice presented a CR/UR ratio of 1.15 ± 0.11 for delay and 1.01 ± 0.1 for trace paradigms, while Lurcher mice presented a ratio of 1.26 ± 0.22 for delay and 1.07 ± 0.2 for trace paradigms. CR/UR ratios reached maximum values during the third conditioning session, when WT values were 0.98 ± 0.05 for delay and 0.8 ± 0.04 for trace paradigms, and Lurcher values were 0.66 ± 0.04 for delay and 0.57 ± 0.06 for trace paradigms. During conditioning, one-way ANOVA showed significant differences between WT and Lurcher mice for delay paradigm in the second [F_(1, 17)_ = 13.3; *P* < 0.01], third [F_(1,17)_ = 12.1; *P* < 0.01], and fourth [F_(1,17)_ = 30.17; *P* < 0.001] sessions, and for trace paradigm in the third [F_(1,17)_ = 8.09; *P* < 0.05] and fourth [*P* < 0.05, Student-Newman-Keuls Method, ANOVA on Ranks] ones. Finally, during extinction sessions, WT mice reached ratios of 1.08 ± 0.06 for delay and 0.9 ± 0.08 for trace paradigms, and Lurchers 1.07 ± 0.13 for delay and 0.8 ± 0.07 for trace paradigms (Fig. 4c,d).

### Timing correlations during CRs of the classical eyeblink conditioning

**Figure 5:**
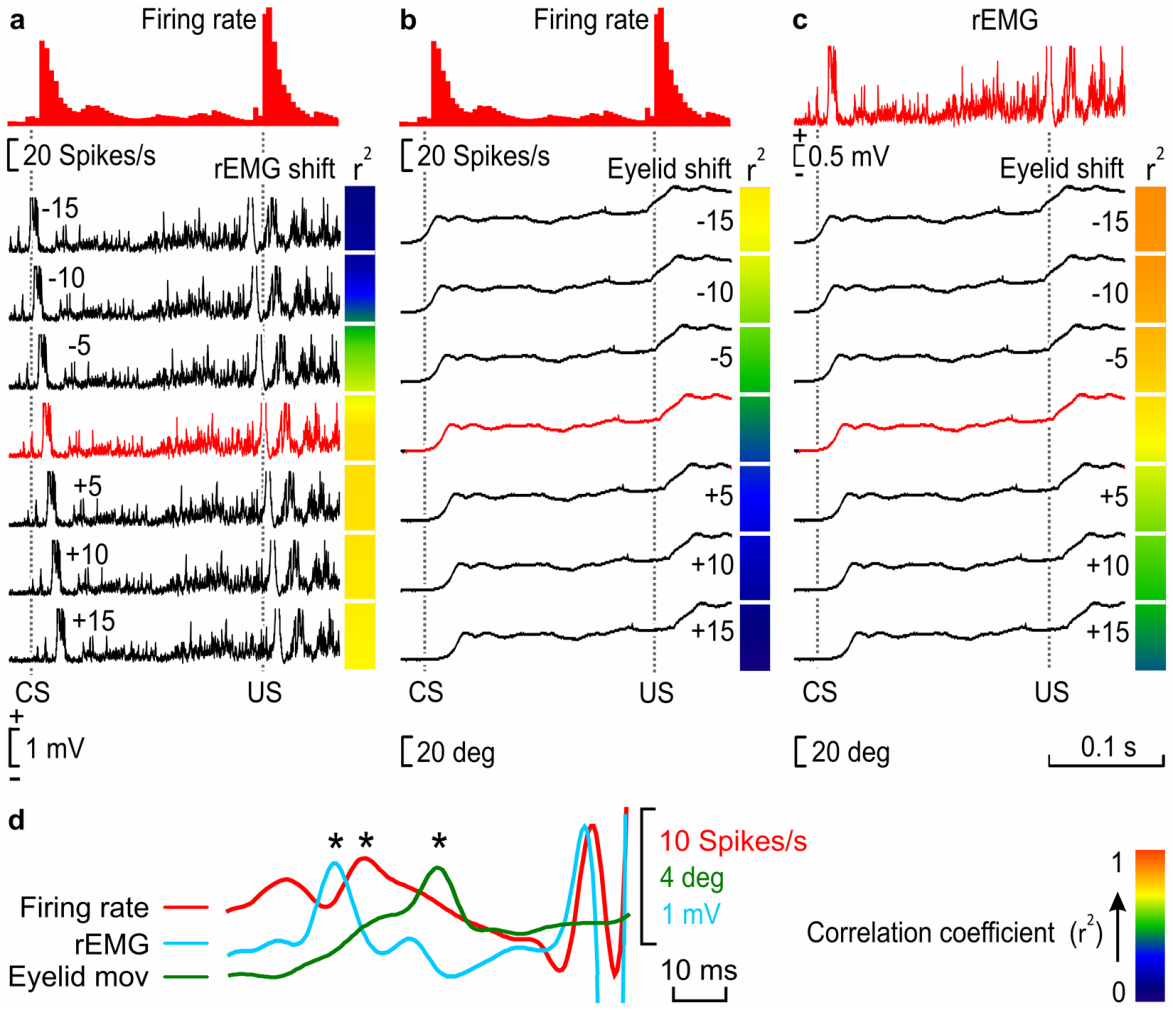
Timing correlation between firing rate, rEMG, and lower-eyelid position for CRs collected during classical eyeblink conditioning. We calculated the coefficient of determination (r^2^) between the firing rate of type A neurons and the rEMG (**a**), the firing rate and eyelid position (**b**), and the rEMG and eyelid position (**c**) in 10 averaged trials. (**a**) A representation of r^2^ values between the firing rate of IPN neurons and the corresponding rEMG (red trace), when the rEMG was shifted −15 ms, −10 ms, −5 ms, +5 ms, +10 ms, and +15 ms (black traces) from the original timing (0 ms, red trace) recorded during a conditioning session of a WT trained mouse during a delayed conditioning paradigm. We compared regions of the CS-(US −20 ms) interval and delimited each one by turning points automatically detected in the firing rate. For each comparison, the collected r^2^ value is represented by a colored square to the right of each rEMG recording. (**b**) Same correlation was calculated for neuron firing rate vs. eyelid position. (**c**) Same correlation is calculated for rEMG vs. eyelid position. The calibration for color gradient values is shown at the bottom right in (**d**). Higher r^2^ values corresponding to negative-shifted recordings indicate a delay with respect to the correlated recording (in **a** and **b**, the firing rate, and in **c**, the rEMG), whereas higher r^2^ values corresponding to positive-shifted recordings indicate an advance with respect to the correlated recording. (**d**) Superimposition of smoothed recordings of the firing rate, rEMG, and eyelid movement, corresponding to the last 80 ms of the CS-US interval of the unshifted recordings shown from (**a**) to (**c**), showing the timing appearance of bursts of the CR (*). Note that, whereas eyelid movement is delayed with respect to the firing rate (**b**) and, obviously, with respect to the rEMG (**c**), interestingly the rEMG is advanced with respect to the firing rate (**a**).

A timing correlation analysis was designed to detect precise temporal differences between the firing rate of IPN type A neurons, the rEMG, and eyelid movements during the performance of eyelid CRs. For that, pairwise comparisons were made between firing rates vs. rEMG activities, firing rates vs. eyelid movements, and rEMG activities vs. eyelid responses (Fig. 5a–c). Then, 6 new channels, copies of one of each pair of the compared channels (rEMG in Fig. 5a, and eyelid movements in Fig. 5b,c), were created and shifted −15 ms, −10 ms, −5 ms, +5 ms, +10 ms, and +15 ms from their original (0 ms) timing. A correlation study was carried out to detect which of them reached a higher coefficient of determination (r^2^) with respect to the compared channel of each pair. A colorimetric gradient was assigned to r^2^ values to facilitate a visual observation of the best detected correlations. In the example illustrated in Fig. 5b, corresponding to a trained WT mouse, the best correlation was detected between the neuron firing rate and the −15 ms-shifted rEMG channel, which means that rEMG events occur ≈15 ms before firing rate ones. As a control of the effectivity of the analysis, we used the comparison between rEMG vs. eyelid movement (Fig. 5c), because muscle activity must precede eyelid displacement. In that example, r^2^ values, which are higher for the −15 ms- and −10 ms-shifted eyelid channels, indicate that eyelid movements occur ≈10–15 ms after rEMG events.

In the same way, Fig. 5b shows that eyelid movements occur after firing rate IPN neurons, although all r^2^ values of the eyelid original and shifted channels correlations indicate a poor correlation (see the color code bar). Finally, a superimposition of smoothed recordings of the firing rate, rEMG, and eyelid movement, corresponding to the last 80 ms of the CS-US interval of the unshifted recordings shown from a to c, is presented in Fig. 5d, showing the timing appearance of neural bursts of the CR (Fig. 5d, asterisks).

On the whole, whereas eyelid positions are delayed with respect to the firing rate of the type A neuron (b) and, obviously, with respect to the rEMG activity of the orbicularis oculi muscle (c), the rEMG of the orbicularis oculi is advanced with respect to the neuronal firing rate (a).

### Timing correlations between neuronal firing, muscle activation, and eyelid position during delay and trace conditioning of WT and Lurcher mice

**Figure 6:**
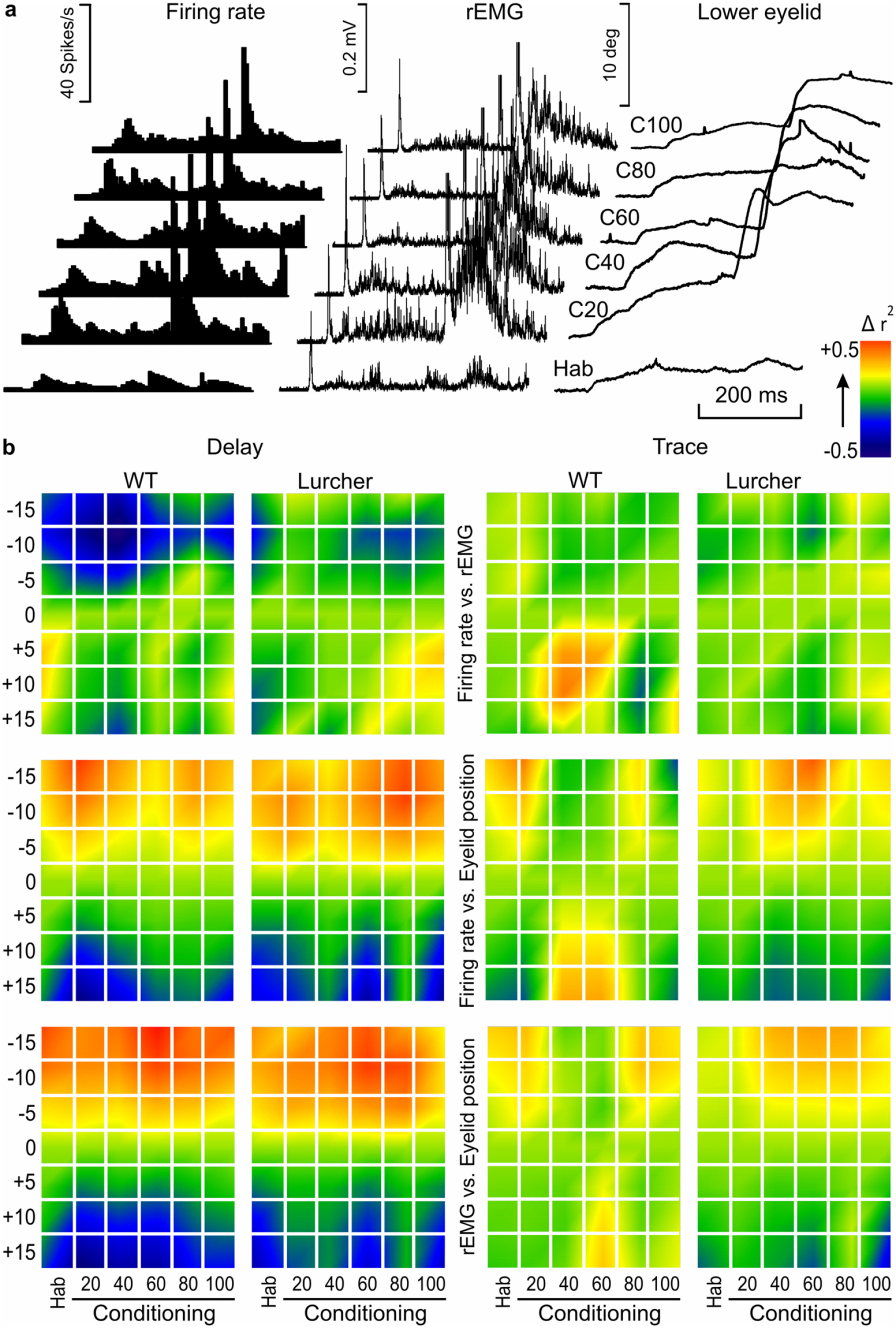
Timing between firing rate, rEMG, and lower-eyelid position during classical eyeblink conditioning using delay and trace paradigms in WT and Lurcher mice. (**a**) A raster representation of firing rate, rEMG, and lower-eyelid position corresponding to the average of 20 habituation (Hab) and 100 conditioning (**c**) trials collected during a single conditioning session. Conditioning trials were averaged in groups of 20. Represented data correspond to a WT mouse conditioned with a delay paradigm. (**b**) Colorimetric representation of the timing correlation between firing rate vs. rEMG (top), firing rate vs. eyelid position (middle), and rEMG vs. eyelid position (bottom) during a single conditioning session from WT and Lurcher mice. r^2^ values were computed between the first and the second set of records, the latter being shifted −15 ms, −10 ms, −5 ms, +5 ms, +10 ms, and +15 ms from the original (0 ms) timing. A comparison was carried out between regions in the CS-(US −20 ms) interval, each one delimited by turning points automatically detected on the first recording. Normalized r^2^ values are represented by colored squares corresponding to each shifted time against each average of training (Hab, Conditioning) trials, mice (WT, Lurcher) types, and conditioning (Delay, Trace) paradigms. The color code of normalized r^2^ values is represented at the top right. Illustrated data correspond to the average of n = 6 animals. Higher r^2^ values corresponding to negative-shifted recordings indicate a delay of the second recording with respect to the first (correlated) one, while higher r^2^ values corresponding to positive-shifted recordings indicate an advance of the second with respect to the first. Thus, it is important to note that whereas eyelid movement is delayed with respect to the firing rate (middle) and, obviously, with respect to the rEMG (bottom), the rEMG is advanced with respect to the firing rate (top), particularly in the case of WT mice conditioned with delay paradigms.

Antidromically identified IPN type A neurons (Fig. 1b) recorded during a complete conditioning of both delay and trace paradigms were used in this analysis. The aim was to quantify changes in the coefficient of determination between neuronal firing rate, rEMG activity of the orbicularis oculi, and eyelid position across the learning process. For that, mice were trained during 20 habituation and 100 conditioning trials, which were averaged in groups of 20 trials. Figure 6a shows raster averages of habituation (HAB) and conditioning (C20-C100) trials, corresponding to firing rate (left), rEMG (middle), and lower-eyelid position (right) channels of a representative WT animal. Note that the best performance of CRs was located between C40 and C60 trials, indicating a relative fatigue taking place during the following conditioning trials.

For timing correlations, all of these recordings were compared as described above (see Fig. 6b). Thus, trial averages of habituation (20) and conditioning (100 in groups of 20) phases of the experiment, corresponding to neuronal firing rate, rEMG activity, and eyelid movements of WT and Lurcher mice conditioned with trace and conditioned paradigms, were compared in pairs (firing rate vs. rEMG, firing rate vs. eyelid responses, and rEMGs vs. eyelid movements). Higher r^2^ values corresponding to negative-shifted recordings indicate a delay of the second with respect to the first (correlated) recording, while higher r^2^ values corresponding to positive-shifted recordings indicate an advance of the second with respect to the first. Thus, whereas eyelid movement is delayed with respect to the firing rate (middle) and, obviously, with respect to the rEMG (bottom), it is important to note that the rEMG is advanced with respect to the firing rate (top), particularly in the case of WT mice conditioned with a delay paradigm, indicating that the EMG activity of the orbicularis oculi preceded the firing rate of IPN type A neurons. Thus, the role of IPN neurons must be related more with the performance of the conditioned eyelid responses than with its acquisition. Furthermore, timing correlations between firing rates and rEMG or eyelid position denote fine differences between paradigms and groups of mice.

## Discussion

Although the neural mechanisms underlying eyeblink conditioning–mostly the delay paradigm–have been delineated more completely than for any other type of mammalian learning, there are a number of critical issues that require further investigation^41^. It is still under discussion whether the cerebellum is the place where this type of associative learning is acquired and stored^42^ or if this structure is mainly involved in the proper performance of learned motor responses acquired in other brain sites^10,43,44^. In the present study, we have tried to approach this question using three different procedures at the same time to determine eyelid dynamics in alert behaving mice: i) a Hall-effect sensor to determine lower-eyelid position^45^; ii) EMG recordings of the activity of the orbicularis oculi muscle; and iii) a high-speed camera analysis to detect the kinematics of eyelid closing. In addition, we have recorded the unitary activity of IPN type A neurons^37,43^ during classical eyelid conditioning using both delay and trace paradigms. Finally, and in order to determine the contribution of cerebellar cortex to the acquisition, storage, and/or performance of eyeblink CRs, we have used both WT and Lurcher animals^21,26–31,35^. We have determined the proper performance of acquired CRs as an index between the rEMG areas corresponding to the CS-US interval and the one presented for the same duration following US presentation. Changes evoked in this performance index were indicative of the relative amplitude and duration of classically conditioned eyelid responses^21^. Another important issue regarding the cerebellar contribution to the initiation of eyeblink CRs is related to the beginning of the acquired activity in IPN type A neurons and the beginning of evoked CRs^21,37,43^. For this, we have analyzed timing correlations between the movement of the eyelid, the EMG of the orbicularis oculi, and the firing rate of IPN type A neurons, by means of an automatic process developed for this study.

Our main results suggest that in comparison with WT mice, Lurcher animals presented an improper performance of acquired CRs during both delay and trace paradigms. In addition, Lurcher mice presented a longer delay in the firing of IPN type A neurons with respect to the orbicularis oculi muscle activation during the performance of CRs. IPN type A neurons in WT mice also lagged the initiation of CRs, but with lower delays. Altogether, the present data suggest that the IPN cannot be considered the place where CRs are generated, but it clearly seems to be involved in its proper performance.

At the same time, we have to take into account other details of our results that, in conjunction with other findings, could contribute to a better understanding of the learning processes taking place during classical conditioning. Thus, in our work, we have found different patterns of eyelid closing for delay and trace conditioning paradigms, although the shapes of those CRs were similar in WT and Lurcher mice. That shape was almost identical to those well described before in mice for delay^15^ and trace^46,47^ conditioning paradigms at early stages of the conditioning, when the position of the eyelid was recorded with technical procedures like the ones used here. Similar results for CR profiles were reported years ago in behaving cats^39^.

The analysis of eyeblink CRs showed differences between WT and Lurcher mice for both delay and trace paradigms, with an impaired performance for the latter. As far as we know, no other analysis of this kind of conditioning has been made based on the calculation of the CR/UR ratios. Other analyses, based on the quantification of CRs^21,35^, and/or CR amplitude^22^, failed to determine these differences between groups. We hypothesize that the physiological and behavioral hyperactivity and motor disinhibition described previously in Lurcker mice^21,23,48^ may contribute to the peculiar profile and amplitude of their CRs. Since URs could also be affected by such disturbances, the relationship between the two motor responses (i.e., the CR/UR performance index) could be used as a control factor for the correct analysis of these eyelid responses.

Although we consider the procedure followed here very suitable for properly identifying IPN neurons by means of their antidromic activation from the red nucleus followed by the collision test^35,37,38,43,44^, it is difficult to record the same neuron and follow its changes in firing rate across successive daily sessions using glass pipettes. For this reason, we aimed to design a single long-lasting conditioning session for the proper study of timing correlation between the three variables included in the present study: unitary activity of IPN type A neurons, EMG activity of the orbicularis oculi muscle, and eyelid position during both CRs and URs. In fact, this experimental problem has previously been approached in other studies of classical eyelid conditioning when, in certain instances, a single conditioning session might be desirable to avoid the unwanted effects of inter-session forgetting^49^, for assessing the effects of single doses of a drug^50^, or for studying the time course of conditioned facilitation of URs^51^. In any case, different classical conditioning experiments have described learning formation at early stages of training in behaving mice^15,52^.

The eyelid motoric is controlled by a push-pull system formed essentially by the orbicularis oculi and the levator palpebrae muscles^44^. As shown here type A neurons projects to the contralateral red nucleus and reinforce the activity of rubral neurons projecting to the contralateral facial motoneurons for the proper performance of CRs^35,37^. Type B neurons are preferentially activated from the oculomotor area and it is assumed that its role is to disfacilitate the activity of the levator palpebrae during eyelid closing^8,14,35^. Purkinje cells also seem to participate in this push-pull system, because they are activated or silenced during the CS-US interval^44,53^. It can be confidently assumed that those Purkinje cells that are activated during the CS-US interval projects on the underlying type B cells, while those that pause during this interval project on type A neurons.

In conclusion, results obtained by performing a timing correlation analysis helped us to confirm the enhancer role of IPN type A neurons in the performance of eyelid CRs using both delay and trace conditioning paradigms^6,7,14,19,54^ but not in the initiation and/or storage of acquired memories. This proposal is further supported by the results collected here with Lurcher mice, indicating that the absence of Purkinje cells modified the performance of CRs and URs, but did not prevent the acquisition of conditioned eyelid responses^35^, and by a recent report^10^ indicating that motor cortex pyramidal cells start firing during the CS-US interval well in advance to cerebellar neurons.

## Methods

### Experimental subjects

We used adult (3–5 months old) WT and mutant (Lurcher) male mice on a B6CBA background. WT and Lurcher mice were obtained from Dr. F. Vožeh’s Laboratory (Charles University, Faculty of Medicine in Pilsen Animal House, Pilsen, Czech Republic) and came from the same litters. Upon their arrival at the Pablo de Olavide Animal House (Seville, Spain), animals were housed in common cages (n ≤ 10 per cage), but after surgery they were housed individually. Mice were kept on a 12:12 h light-dark cycle with constant ambient temperature (21 ± 1 °C) and humidity (50 ± 7%). Food and water were available *ad libitum*. Animals were divided into two experimental groups. A total of 9 WT and 9 Lurcher were included in the first group and 6 WT and 6 Lurcher in the second group, for each trace and delay paradigms.

### Ethics statement

All experiments were carried out in accordance with the guidelines of the European Union Council (2010/276:33–79/EU) and Spanish regulations (BOE 34:11370-421, 2013) for the use of laboratory animals in chronic experiments. Experiments were also approved by the local Ethics Committee of Pablo de Olavide University (Seville, Spain).

### Surgery

Animals were anesthetized with a mixture of Ketamine (35 mg/kg) and Xylazine (2 mg/kg) i.p. All mice were implanted with four electrodes in the upper eyelid of the left eye. Electrodes were made from Teflon-insulated, annealed stainless steel wire (50 µm in diameter, A-M Systems, Carlsborg, WA, USA). One pair of electrodes was aimed towards the supraorbital nerve for the application of electrical stimuli. The second pair of electrodes was implanted in the ipsilateral orbicularis oculi muscle for recording its EMG activity. The four electrodes were connected to a 4-pin socket (RS-Amidata, Madrid, Spain) which was affixed with dental cement to the cranial bone. The surgical procedure is schematized in Fig. 1a.

Animals of the second group were also implanted with a holding system, consisting of a head-plate, designed for its implantation to the arm of a stereotaxic apparatus. The plate was fixed to the skull with the help of two small screws and dental cement. Animals were placed in a stereotaxic device (David Kopf Instruments, Tujunga, CA, USA), and the bregma-lambda axis of the skull was situated in the horizontal plane, parallel to the rostro-caudal axis; the aim was to stabilize the head during the next surgical steps, as well as during the following experiments. Then, a craniotomy was carried out in the occipital skull to allow recording the unitary activity of IPN neurons during conditioning. A recording chamber was built around the craniotomy with acrylic cement and, finally, the chamber was covered with sterile gauze and bone wax until the recording sessions. A silver electrode (1 mm in diameter), in contact with the dura mater, was attached to the left parietal bone as a ground. A 1-mm diameter hole was made in the skull, over the underlying contralateral red nucleus and covered with bone wax. In a second surgical step, behaving mice were fixed by their head-plate to the holding system, and implanted in the red nucleus (stereotaxic coordinates: AP, −3.52; L, −0.62; and D, 3.7)^36^ with a pair of stimulating electrodes made from 50 µm, Teflon-coated tungsten wire (Advent Research Materials Ltd, Eynsham, England), using the hole previously made. The final position of these stimulating electrodes was determined by eyelid-closing movements evoked by pairs of pulses (1 ms interpulse interval) applied in the red nucleus. Electrodes were soldered to a 2-pin socket and fixed to the skull with acrylic cement.

### Classical conditioning procedures

For classical eyeblink conditioning, animals of the first group were placed individually in small (15 cm × 5 cm × 10 cm) methacrylate cages, which were covered with a ground-connected metallic sheet, to eliminate electrical interferences. The cages allowed adequate ventilation and the passage of a 4-band wire connecting the animal to the recording/stimulating system.

Both delay and trace conditioning paradigms were carried out. For this, animals were presented with a tone (6000 Hz and 95 dB, lasting 250 ms for delay and 20 ms for trace) as a CS, followed 250 ms later by an electrical stimulation (500 µs, 3 × Threshold) as a US. CS-US presentations were separated at random by 30 ± 5 s. For habituation and extinction sessions, only the CS was presented, also at intervals of 30 ± 5 s. During five days, 200 trials, divided into four 50-trial sessions, were presented to each animal. Distribution of habituation, conditioning, and extinction sessions is schematized in Fig. 4a.

The EMG activity of the orbicularis oculi muscle was recorded using differential amplifiers with a bandwidth of 1 Hz to 10 kHz (Grass Technologies, West Warwick, USA). Data were stored directly on a computer through an analog/digital converter (CED 1401 Plus, Cambridge Electronic Design, Cambridge, England), at a sampling frequency of 11–22 kHz and an amplitude resolution of 12 bits. Data collected from the first experimental group during the five training sessions were analyzed off-line for quantification of EMG responses with the help of the Signal Average Program (Cambridge Electronic Design).

CR/UR ratios were calculated from collected EMG recordings. For this, the rectified and filtered (250 Hz, high pass) EMG area (in mV × s) collected from the CS-US interval (i.e., 250 ms) was compared with one of an equal duration, starting from the US presentation. A 10-ms interval around the US artifact and the first 50-ms of the CS-US interval (see Fig. 4b) were excluded from the analysis. Data were normalized assigning value = 1 to the CR/UR of the third habituation (H3) session.

Statistical analysis was carried out using the Sigma Plot 11.00 software (Systat Software, Inc. Germany), for a significance level of *P* < 0.05. Mean values are followed by their SEM. A one-way ANOVA was calculated comparing the different session of both groups and, when indicated, a pairwise comparison procedures of the Student-Newman-Keuls method was performed.

### Recording and stimulating procedures

In the second series of experiments, we recorded the unitary activity of antidromically identified IPN neurons during classical conditioning of eyelid responses. For that, each mouse was fixed by its head-plate to the bars of a stereotaxic apparatus, while their legs rested on a muffled running wheel adapted to the animal. The recording room was softly illuminated during the experiments and provided with a 60-dB background white noise. The experiment began after a few sessions during which the mouse was accustomed to walking on the running wheel with its head fixed to the bars. The EMG activity of the orbicularis oculi muscle was recorded as described above. Neuronal unitary activity was recorded with the help of an AM 3000 AC/DC differential amplifier (A-M Systems, Inc., Carlsborg, USA). Unitary recordings were performed with glass micropipettes filled with 3 M NaCl (2–5 MΩ of resistance) coupled to a preamplifier, and filtered in a bandwidth of 1 Hz to 25 kHz. The recording area was approached with the help of stereotaxic coordinates^36^, and antidromic field potentials were evoked by electrical stimulation of the contralateral red nucleus. Electrical stimulation of the red nucleus consisted of paired (cathodic, 500 µs, <0.6 mA, 5-ms to 200-ms interval) pulses programmed with a CS-220 stimulator across an ISU-220 isolation unit (Cibertec, Madrid, Spain). Criteria were systematically followed to determine whether the recorded and the activated neurons were the same^38,55^. At the end of each recording session, the recording micropipette was removed and the recording chamber sterilized and closed with bone wax. Eyelid movements were recorded as the voltage difference between a Hall-effect sensor and a magnetic cylinder (1.2 mm Ø, 0.5 mm height) fixed to the lower eyelid. Maximum angular displacements of the lower eyelid were ≈30° for all the animals. For the sake of homogeneity, the gain of the recording system was adjusted to yield 1 V per 10°.

Before experiment onset, air puffs were applied to the eyes, to record eyeblink-related IPN neurons. Recorded neurons were classified as air-puff-activated (type A) or air-puff-inhibited (type B) neurons (Fig. 1c–d). For further correlations between IPN neurons, EMG, and eyelid movement recordings during the CRs, only type A neurons were used.

For the analysis of eye opening, a total of 100 photographs (1 photograph/5 ms) of the eye were made with a fast charge-coupled device (CCD) camera (Pike F-032, Allied Technologies, Stadtroda, Germany). Photographs were taken for 500 ms, from 50 ms before CS presentation until 450 ms after it, and quantified off-line with a homemade MATLAB-based program (Fig. 1e–f). A total of 20 habituation and 100 conditioning trials, divided for analysis in groups of 20, were presented to each animal.

### Data collection and analysis

Unitary activity of IPN neurons, unrectified EMG activity of the orbicularis oculi muscle, lower-eyelid position, and 1-V rectangular pulses corresponding to CS, US, and electrical stimulations presented during the different experiments were stored digitally on a computer through an analog–digital converter (1401- plus, Cambridge Electronic Design) for quantitative off-line analysis. Collected data were sampled at 25 kHz for unitary recordings or at 10 kHz for EMG and eyelid-position recordings, with an amplitude resolution of 12 bits. A computer program (Spike2, Cambridge Electronic Design) was used to display single and overlapping representations of unitary activity, EMG activity of the orbicularis oculi muscle, and eyelid position.

In most of the cases, recorded IPN neurons were easily isolated from other neurons during experimental recording sessions. Wherever it was not possible to isolate and record a single cell, a spike sorting (Spike2, Cambridge electronic Design) was performed to classify the different recorded neurons. The spike-sorting analysis was performed off-line by creating a new wave-mark based on the raw neuronal recording. Amplitude and duration thresholds were adjusted to include all types of spike while excluding the recorded noise. The spike duration threshold was set at 0.5 ms and the amplitude threshold at 0.1 mV, depending on the noise level from each recording session. Templates created by the spike-sorting program were meticulously examined, and those presenting non-physiological signals were eliminated. Finally, an event channel for the selected neuron was created in which each event corresponded to a spike. The instantaneous firing rate was automatically calculated from this channel with the help of the Spike 2 software.

### Correlation analysis

For events average and the analysis of putative correlations between neuronal firing rates, rEMG profiles, and eyelid positions, all data collected with the Spike 2 program were exported to the Signal 5.11 software (Signal, Cambridge Electronic Design). Next, new channels, copying the selected rEMG file, were created and shifted −15 ms, −10 ms, −5 ms, +5 ms, +10 ms, and +15 ms from the original timing, and, with the help of two active cursors, segments of the CS-US interval were delimited by turning points automatically detected on the firing-rate channel, and the area of each of them was calculated (Fig. 5a) and stored. Then, and with the help of Sigma Plot 11.00 software, we carried out a global analysis of linear correlations between those IPN-neuron firing-rate segment areas, and the segment areas corresponding to the original and the shifted rEMG channels. The same procedure was used to calculate the coefficients of determination between neuronal firing rates and lower-eyelid positions (Fig. 5b) and between rEMG profiles and lower-eyelid positions (Fig. 5c). A 20-ms interval, corresponding to US artifacts of original and shifted channels, was excluded from the analysis.

For the analysis of the single-session conditioning, the coefficient of determination (r^2^) was calculated from averages of groups of 20 events (20 habituation and 5 groups of 20 conditioning events), and the r^2^ values of n = 6 animals were normalized and averaged to obtain the final normalized r^2^, which was represented by colorimetric gradients (Fig. 6b).

### Histological study

At the end of the behavioral studies, mice were re-anesthetized with Ketamine (35 mg/kg) and chloral hydrate (4%), and perfused transcardially with saline and 4% phosphate-buffered paraformaldehyde. Selected sections (40 µm) including the red nucleus and cerebellar nuclei were mounted on gelatinized glass slides and stained using the Nissl technique with 0.1% cresyl violet, to determine the proper location of stimulating electrodes and recording micropipettes. Photomicrographs were taken with the help of a 5x lens of a Leica DMRE microscope equipped with a Leica DFC550 camera, and with the LAS V4.2 software (Leica Microsystems GmbH, Wetzlar, Germany). Photomicrograph reconstructions were made with the Microsoft Office Professional Plus 2010 and CorelDRAW X4 software.

### Data availability

The datasets generated during and/or analyzed during the current study are available from the corresponding author on reasonable request.
